# InterLabelGO+: unraveling label correlations in protein function prediction

**DOI:** 10.1093/bioinformatics/btae655

**Published:** 2024-11-05

**Authors:** Quancheng Liu, Chengxin Zhang, Lydia Freddolino

**Affiliations:** Department of Computational Medicine and Bioinformatics, University of Michigan, Ann Arbor, MI, 48109, USA; Department of Computational Medicine and Bioinformatics, University of Michigan, Ann Arbor, MI, 48109, USA; Department of Biological Chemistry, University of Michigan, Ann Arbor, MI, 48109, USA; Department of Computational Medicine and Bioinformatics, University of Michigan, Ann Arbor, MI, 48109, USA; Department of Biological Chemistry, University of Michigan, Ann Arbor, MI, 48109, USA

## Abstract

**Motivation:**

Accurate protein function prediction is crucial for understanding biological processes and advancing biomedical research. However, the rapid growth of protein sequences far outpaces the experimental characterization of their functions, necessitating the development of automated computational methods.

**Results:**

We present InterLabelGO+, a hybrid approach that integrates a deep learning-based method with an alignment-based method for improved protein function prediction. InterLabelGO+ incorporates a novel loss function that addresses label dependency and imbalance and further enhances performance through dynamic weighting of the alignment-based component. A preliminary version of InterLabelGO+ achieved a strong performance in the CAFA5 challenge, ranking sixth out of 1625 participating teams. Comprehensive evaluations on large-scale protein function prediction tasks demonstrate InterLabelGO+’s ability to accurately predict Gene Ontology terms across various functional categories and evaluation metrics.

**Availability and implementation:**

The source code and datasets for InterLabelGO+ are freely available on GitHub at https://github.com/QuanEvans/InterLabelGO. A web-server is available at https://seq2fun.dcmb.med.umich.edu/InterLabelGO/. The software is implemented in Python and PyTorch, and is supported on Linux and macOS.

## 1 Introduction

With rapid advancements in high-throughput sequencing technologies, the space of known protein sequences has expanded at an unprecedented rate. Despite this surge in sequence data, our ability to experimentally determine protein functions remains significantly limited. This discrepancy has led to a growing divide between the vast number of identified sequences and the relatively small subset with experimentally confirmed functions (Friedberg 2006). To address this challenge, it is crucial to develop automated computational methods capable of accurately predicting protein functions on a large scale.

Many computational approaches have been developed to address the protein function prediction problem. Sequence template-based methods, such as GOtcha (Martin *et al.* 2004) and Blast2GO (Götz *et al.* 2008), rely on sequence similarity to transfer functional annotations from well-characterized proteins to query sequences. Structural template-based methods, such as COFACTOR (Zhang *et al.* 2017) and ProFunc (Laskowski *et al.* 2005), utilize structural information to infer protein functions. However, these methods have limitations in capturing distant evolutionary relationships and struggle with proteins that lack close homologs with known functions. Literature-based methods, such as DeepText2GO (You *et al.* 2018), aim to extract functional information from the abstracts of protein-related publications using natural language processing techniques. These methods can capture valuable functional insights from unstructured text data but face challenges of information retrieval accuracy, and the availability of relevant literature for all proteins. Deep learning-based methods, such as DeepGOPlus (Kulmanov and Hoehndorf 2020) and TALE (Cao and Shen 2021), extract sequence embeddings using convolutional neural networks and transformers, respectively, to predict protein functions. While these methods can capture complex patterns in protein sequences, they may not fully exploit the rich evolutionary information contained in protein language models (PLMs).

To overcome the limitations of existing methods and address the large-scale and multi-label nature of protein function prediction, we developed InterLabelGO+, a hybrid approach that integrates InterLabelGO, a deep learning-based method leveraging PLMs, with AlignmentKNN, an alignment-based method, for improved performance. InterLabelGO, the core deep learning component of InterLabelGO+, incorporates a novel loss function that integrates label dependency and addresses label imbalances, enabling the model to capture complex functional relationships and handle the skewed distribution of functional annotations. InterLabelGO+ further enhances this approach by incorporating AlignmentKNN through a dynamic weighting scheme based on the mean top sequence identity. This allows the model to effectively leverage both the complex patterns captured by deep learning and the evolutionary information contained in sequence alignments. A preliminary version of InterLabelGO+ participated in the Critical Assessment of Functional Annotation 5 (CAFA5) challenge (Zhou *et al.* 2019; https://www.kaggle.com/competitions/cafa-5-protein-function-prediction/leaderboard) and achieved a strong performance, ranking sixth out of 1625 participating teams. This demonstrates the efficacy of our approach in comparison to state-of-the-art methods.

## 2 Materials and methods

### 2.1 The overall framework of InterLabelGO+

**Figure 1.**
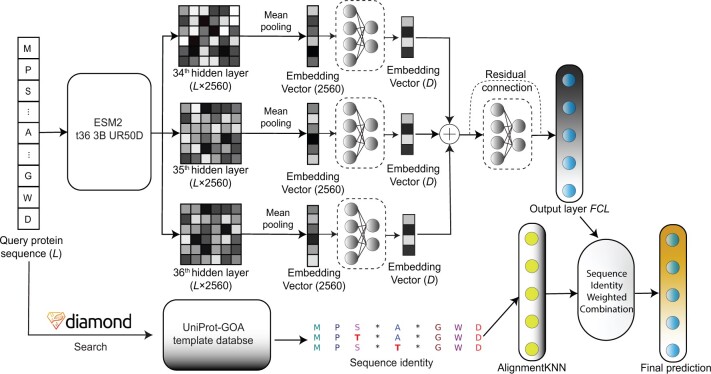
The workflow of InterLabelGO+. Throughout the manuscript, we use “InterLabelGO” to refer to the top (deep learning) branch of the pipeline, and “AlignmentKNN” to refer to the (alignment-based) bottom branch.

The overall layout of the hybrid InterLabelGO+ is shown in Fig. 1. InterLabelGO, the deep learning component, begins with inputting the query protein sequence into the ESM2 (Verkuil *et al.* 2022) model, which generates a sequence embedding matrix from the last three hidden layers, with each layer producing a 3×L×2560 matrix where *L* represents the protein sequence length. Mean pooling is applied to the embedding vectors corresponding to each residue, resulting in compressed embedding vectors of length 2560 from each hidden layer. Three parallel multilayer perceptrons (MLPs) further processed these vectors representing embeddings. Each MLP is responsible for extracting evolutionary features from its corresponding layer, resulting in a 3 × 2560 matrix. These aggregated evolutionary data are then concatenated and processed by another MLP block. This layer’s purpose is to transform the ESM2-derived features into GO term probabilities. During the inference phase, a hierarchical post-processing approach is implemented, mandating that the probability of a parent term is at least equal to the maximum probability of its child terms, and raising the probabilities assigned to all non-leaf nodes to bring the predictions in line with this constraint.

In addition to the deep learning-based predictions from InterLabelGO, InterLabelGO+ also incorporates homology-based function transfer using AlignmentKNN, which is adapted from Zhang and Freddolino (2024). This approach involves searching the query sequence against a database of annotated template proteins using DIAMOND (Buchfink *et al.* 2021) and deriving prediction scores from normalized bitscores and sequence identity (detailed in Section 2.3). The final predictions of InterLabelGO+ are obtained by integrating the deep learning-based predictions with the homology-based predictions using a weighted combination approach (described in Section 2.4). This amalgamation of homology-based and deep-learning predictions provides the final output for protein function prediction.

### 2.2 Loss in multi-label classification

In the context of protein function prediction using GO annotations, we employ a composite loss function that addresses the challenges of class imbalance and captures label dependencies. The composite loss function consists of two components: an F1-score based loss that accounts for class imbalance and a rank-based loss that captures label dependencies. In the following subsections, we will define each component and then present the composite loss function.

#### 2.2.1 F1-score based loss addressing class imbalance

A significant challenge in protein function prediction using GO annotations arises from the label imbalance among GO terms. This imbalance, where certain terms are over-represented and others are rare, can lead to suboptimal performance in standard loss functions, such as binary cross-entropy (BCE), as less frequent terms do not contribute sufficiently to the overall loss function.

To overcome this challenge, our approach incorporates a specialized F1 loss function, enhanced with the Information Accretion (IA) weight. IA, also known as Information Content, as introduced in Clark and Radivojac (2013), quantifies the additional information a term *q* contributes to an ontology annotation, assuming its parent nodes are already annotated. It is calculated as:

(1) $$\text{IA}(q)={\text{log}\;}_{2}\left(\frac{1+\left|\text{proteins}\;\text{with}\;\text{parent}\;\text{term}(s)\;\text{of}\;q\right|}{1+\left|\text{proteins}\;\text{with}\;\text{term}\;q\right|}\right),$$

where |X| denotes the cardinality of set *X*.

The IA weight prioritizes GO terms with higher informational values, aiming to yield more informative predictions.

By factoring both precision and recall, the F1 loss function is inherently suitable for scenarios with label imbalance. The inclusion of the IA weight ensures that the function not only maintains a balance between precision and recall but also emphasizes the significance of more informative GO terms in the model’s learning process.

We employ two variants of the F1 loss: protein-centric and GO-centric. For the protein-centric F1 loss, precision and recall values are calculated for each protein in the batch, considering all its associated GO terms. In contrast, for the GO-centric F1 loss, precision and recall values are calculated for each GO term across all proteins in the batch.

For a single protein *i*, the precision and recall are calculated as follows:

(2) $${\text{Precision}}_{i}=\frac{\sum_{q=1}^{Q}y_{\text{true}}(i,q)\cdot y_{\text{pred}}(i,q)\cdot \text{IA}(q)}{\epsilon +\sum_{q=1}^{Q}y_{\text{pred}(i,q)}\cdot \text{IA}(q)},$$

(3) $${\text{Recall}}_{i}=\frac{\sum_{q=1}^{Q}y_{\text{true}}(i,q)\cdot y_{\text{pred}}(i,q)\cdot \text{IA}(q)}{\epsilon +\sum_{q=1}^{Q}y_{\text{true}}(i,q)\cdot \text{IA}(q)}.$$

For a single GO term *q*, the precision and recall are calculated as follows:

(4) $${\text{Precision}}_{q}=\frac{\sum_{i=1}^{I}y_{\text{true}}(i,q)\cdot y_{\text{pred}}(i,q)\cdot \text{IA}(q)}{\epsilon +\sum_{i=1}^{I}y_{\text{pred}(i,q)}\cdot \text{IA}(q)},$$

(5) $${\text{Recall}}_{q}=\frac{\sum_{i=1}^{I}y_{\text{true}}(i,q)\cdot y_{\text{pred}}(i,q)\cdot \text{IA}(q)}{\epsilon +\sum_{i=1}^{I}y_{\text{true}}(i,q)\cdot \text{IA}(q)}.$$

The F1 loss for both protein-centric and GO-centric variants is calculated as:

(6) $${F1\;\text{Loss}}_{x}=1-\frac{2\cdot {\text{Precision}}_{x}\cdot {\text{Recall}}_{x}}{{\text{Precision}}_{x}+{\text{Recall}}_{x}},$$

where *x* can be *i* (for protein-centric) or *q* (for GO-centric).

In these equations, *I* is the total number of proteins in a batch, and *Q* is the total number of GO terms. $y_{\text{true}}(i,q)$ is the true label for protein *i* and GO term *q*, while $y_{\text{pred}}(i,q)$ is the predicted label for protein *i* and GO term *q*. IA(*q*) is the IA weight for GO term *q*, and *ϵ* is a small constant (1e-16) to avoid division by zero. For the protein-centric F1 loss (${F1\;\text{Loss}}_{i}$), the mean precision and recall values are calculated over the protein dimension *i*. For the GO-centric F1 loss (${F1\;\text{Loss}}_{q}$), the mean precision and recall values are calculated over the GO term dimension *q*.

#### 2.2.2 Rank-based loss capturing label dependencies

The protein GO term prediction problem can be formulated as a hierarchical multi-label classification challenge. The unique challenge here is the structure of the GO term hierarchy, which form a large interconnected network, organized as three Directed Acyclic Graphs (DAGs) for the three GO aspects (MFO, BPO, and CCO). This structure suggests that the prediction for one GO term may be influenced by other terms, acknowledging the inherent correlation and interdependence among them. In general, classification algorithms aim to determine the conditional probability P(y|x), capturing dependencies between input features x and target labels y. In multi-label classification, the complexity increases as dependencies may arise not just between features and target categories, but also among the categories themselves. Conventional methods, such as DeepGO (Kulmanov *et al.* 2018), which typically utilize BCE loss, often overlook the interdependencies between labels when treating each GO term as an independent binary classification problem. To better address these hierarchical and interdependent of GO terms, we adopt the Zero-bounded Log-sum-exp and Pairwise Rank-based (ZLPR) loss (Su *et al.* 2022), shown in Equation (7).

(7) $$L_{\text{zlpr}}=\text{log}\;\left(e^{-s_{0}}+\sum_{i\in \Omega_{\text{pos}}}e^{-s_{i}}\right)+\text{log}\;\left(e^{s_{0}}+\sum_{j\in \Omega_{\text{neg}}}e^{s_{j}}\right).$$

Here, *s_i_* and *s_j_* represent the logits from Output layer *FCL*, as shown in Fig. 1, for the *i*th positive and *j*th negative GO terms, respectively. Ω_pos_ and Ω_neg_ denote the sets of indices for the positive and negative GO terms. The ZLPR loss function introduces a pseudo term *s*_0_, set to 0 in our model, which acts as an intermediate threshold for distinguishing between positive and negative categories. *L*_zlpr_ represents the ZLPR loss for a single protein.

The ZLPR loss function effectively captures the dependencies among GO terms by leveraging information from their joint distribution. The formula distinctly differentiates between positive and negative categories using the log-sum-exp function and the pairwise ranking principle. By minimizing the ZLPR loss, the model aims to maximize the difference between the highest negative logit and the lowest positive logit, effectively ranking the positive categories above the negative ones through the intermediate term *s*_0_. This approach enables the model to consider the relationships between GO terms during the training process, rather than treating each GO term as an independent binary classification problem. Thus, it captures the complex correlations and dependencies among labels, which are crucial for accurate GO term prediction in the context of multi-label classification.

#### 2.2.3 Composite final loss function

To synthesize the strengths of the individual loss components, our final loss function is formulated as a multiplicative combination of the F1 loss calculated on a protein-centric basis, the F1 loss evaluated from a GO term-centric perspective, and the ZLPR loss. This composite approach ensures that the model optimally balances these different aspects during training, leading to enhanced prediction accuracy. The final loss function is defined as:

(8) $$L_{\text{final}}={F1\;\text{Loss}}_{i}\cdot {F1\;\text{Loss}}_{q}\cdot L_{\text{ZLPR}}.$$

Here, ${F1\;\text{Loss}}_{i}$ represents the protein-centric F1 loss averaged across all proteins in the batch, ${F1\;\text{Loss}}_{q}$ denotes the GO term-centric F1 loss averaged for each GO term across all proteins, and $L_{\text{ZLPR}}$ is the mean *L*_zlpr_ per protein, which captures the interdependencies among the GO terms. By integrating these components, the final loss function ensures a comprehensive and balanced learning process, taking into account both the individual and correlation-based characteristics of the protein function prediction task.

A detailed comparison of different loss functions and their combinations, along with their impact on the performance of InterLabelGO, is presented in Section 3.2.

### 2.3 Sequence homology-based AlignmentKNN

Homology-based function transfer is a foundational and extensively utilized method in predicting protein functions. This approach is based on the premise that proteins with similar sequences often exhibit similar functions. Within the AlignmentKNN framework, the sequence of a query protein is searched against the database of annotated template proteins using Diamond. The prediction score is derived from normalized bitscores and sequence identity as follows:

(9) $$S_{\text{align}}(q)=\frac{\sum_{k=1}^{K}{\text{bitscore}}_{k}\cdot {\text{ID}}_{k}\cdot 1(q\in T_{k})}{\sum_{k=1}^{K}{\text{bitscore}}_{k}\cdot {\text{ID}}_{k}},$$

(10) $${\text{ID}}_{k}=\frac{n{\text{ident}}_{k}}{\text{max}(\text{qlen},{\text{slen}}_{k})}.$$

In this equation, *q* represents the *q*th GO term. The terms bitscore_*k*_ and ID_*k*_ denote the bit-score and sequence identity of the *k*th Diamond hit, respectively. The total number of hits considered, *K*, includes those hits which contain at least one GO term from the training set. *T_K_* refers to the set of experimental annotations for the *k*th target protein hit. The function 1 is an indicator function that returns 1 if the GO term *f* is present in *T_K_*, and 0 otherwise.

The sequence identity ID_*k*_ for *k*th protein hit is calculated in Equation (10) where *n*ident represents the number of identical amino acids found in the alignment between the query protein and the target. qlen and slen are denote the lengths of the query protein and the target protein, respectively.

### 2.4 Composite model of InterLabelGO+ using sequence identity

InterLabelGO+ is a weighted combination of AlignmentKNN and InterLabelGO. This approach is distinct from other methods, many of which rely on a linear combination of alignment scores that cannot capture complex relationships between scores, especially in cases where similar proteins are limited in number. InterLabelGO+ incorporates the mean top sequence identity for dynamic weighting, as detailed in Equations (11) and (12).

(11) $$S_{\text{dnn}+}(q)=w\times S_{\text{dnn}}(q)+(1-w)\times S_{\text{align}}(q),$$

(12) $$w=\alpha +(1-\alpha )\cdot e^{-k\cdot I\bar{\text{D}}}.$$

Here, $S_{\text{dnn}+}(q)$ represents the confidence score of InterLabelGO+ for the GO term *q*. The terms $S_{\text{dnn}}(q)$ and $S_{\text{align}}(q)$ denote the confidence scores for term *q* as predicted by InterLabelGO and AlignmentKNN, respectively. The weight parameter *w*, as defined in Equation (12), is calculated using the average of the top five sequence identities, represented by $I\bar{\text{D}}$ in Equation (12). This average is combined with the minimum weight assigned by InterLabelGO, denoted as *α*. Additionally, the parameter *K* determines the rate at which the InterLabelGO weight varies with the average top sequence identities. The optimal values for *α* and *K* were established as 0.33 and 3, respectively, based on evaluations using the validation dataset.

### 2.5 Evaluation

To evaluate our predictions, we adopted the CAFA (Zhou *et al.* 2019) challenge metrics, specifically the maximum weighted F-measure ($wF_{\text{max}}$) and the minimum semantic distance ($S_{\text{min}}$) (Clark and Radivojac 2013). In addition, we report the area under the weighted precision-recall curve (AUWPR), which is particularly relevant for datasets with significant label imbalance as it places greater emphasis on correctly predicting minority classes (Davis and Goadrich 2006). The detailed description of these metrics and their calculations can be found in the Supplementary Materials (SI Section 1).

### 2.6 Datasets

We applied an approach closely paralleling the CAFA experiment for the benchmark datasets construction (Zhou *et al.* 2019). We downloaded all protein sequences from UniProtKB and GO term annotations from the Gene Ontology Annotation (GOA) database in November 2022, March 2023, and February 2024 (Ashburner *et al.* 2000, Huntley *et al.* 2014, Aleksander *et al.* 2023). We then filtered the proteins to include only those with at least one experimental annotation, identified by the following evidence codes: IDA, IPI, EXP, IGI, IMP, IEP, IC, or TAS. These annotations were subsequently propagated within the GO’s hierarchical structure to the root, using only the “is-a” and “part-of” relational criteria. The dataset was chronologically divided into training, validation, and testing subsets as follows: The training dataset comprised proteins annotated up to November 2022. The validation and testing datasets included no-knowledge and limited-knowledge proteins annotated between November 2022 and March 2023 and between March 2023 and February 2024, respectively. No-knowledge proteins are defined as those that lacked any experimental annotations prior to the start of the validation or testing period but gained at least one experimental annotation by the end of the corresponding period. In contrast, limited-knowledge proteins are characterized by having their first experimental annotations in the target GO aspects within the validation or test timeframe, while also possessing experimental annotations in at least one other domain before the start of the period. To optimize the training process and predictive accuracy, we only considered GO terms that met a minimum threshold of training instances. Specifically, we required at least 50 sequences for Biological Process (BPO) terms, and at least 10 sequences for both Molecular Function (MFO) and Cellular Component (CCO) terms. This criterion resulted in the inclusion of 2871 terms for the MFO, 5311 for the BPO, and 1626 for the CCO sub-ontologies, respectively. Detailed statistics on the benchmark datasets are shown on Supplementary Table S1.

## 3 Result

### 3.1 Overall performance of InterLabelGO+

To assess the performance of InterLabelGO+, we compared it with four publicly available state-of-the-art composite models [ATGO+ (Zhu *et al.* 2022), DeepGOPlus (Kulmanov and Hoehndorf 2020), TALE+ (Cao and Shen 2021), and SPROF-GO (Yuan *et al.* 2023)] on the test set (described in Supplementary Table S1). ATGO+ was selected because its architecture inspired InterLabelGO+. DeepGOPlus and TALE+ were selected due to being typical methods that do not use large language models. SPROF-GO was selected due to its good performance reported in CAFA5 (although it was withdrawn prior to the final evaluation). Although there are other high performance methods such as NetGO3 (Wang *et al.* 2023) that placed very highly in CAFA5, they do not have standalone packages and therefore cannot be included for large-scale benchmark here. We evaluated the models using three metrics: *wF*_max_, *S*_min_, and AUWPR. Additionally, we included an alignment-based method (AlignmentKNN) and a naïve baseline model for comparison, the naïve method calculates the confidence score of a query being associated with a GO term by the frequency of that term in the training dataset. For the four third-party methods, we retrained the programs using the same dataset as InterLabelGO+ (detailed in Supplementary Table S1) with their default training parameters and ran them on our test dataset under the default setting.

**Figure 2.**
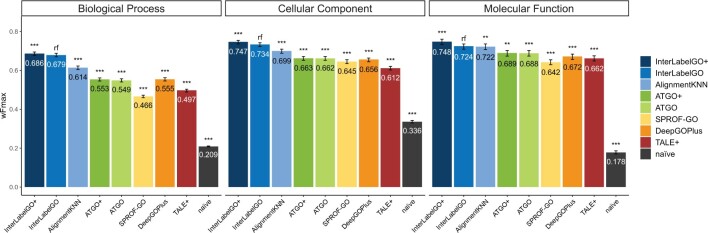
Evaluation of method performance using *w*$F_{\text{max}}$. This single bar plot illustrates the performance of various methods based on the UniprotGOA 202303–202402 release. Each method’s bar is annotated with the *w*$F_{\text{max}}$ value. Statistical significance is indicated by “***” for *P*-values ≤.001, derived from a two-tailed paired *t*-test between InterLabelGO+ and other methods using per-target *w*$F_{\text{max}}$ values. The “rf” sign denotes the reference method. The 95% confidence intervals, shown as error bars, are calculated via 9999 bootstrap iterations on the benchmark dataset.

The results of the comparison are presented in Supplementary Table S2 and Fig. 2. InterLabelGO+ consistently achieves the best predictions for all GO aspects when evaluated by all metrics.

Notably, AlignmentKNN showed complementary performance to neural network-based methods and outperformed other deep learning methods. This observation suggests that as the number of experimental annotations grows in the database, the performance of alignment-based methods may also improve. The complementarity of AlignmentKNN and InterLabelGO is particularly evident in the AUWPR for the MFO sub-ontology (Supplementary Fig. S1C). Although the *wF*_max_ scores for InterLabelGO and AlignmentKNN are similar, their precision-recall curves exhibit slightly different distributions, indicating that these methods capture different aspects of the protein function prediction problem.

The superior performance of our composite InterLabelGO+ method, which leverages the powerful ESM2, compared to the state-of-the-art methods highlights the effectiveness of our approach in capturing the complex relationships between GO terms and protein sequences. Indeed, InterLabelGO with ESM2 outperforms a similar model without ESM2 by 40.9%, 12.8%, and 3.7% higher wFmax for BPO, MFO, and CCO, respectively (Supplementary Fig. S2; n.b. these evaluations were performed using only the deep learning component of our pipeline, not the full composite InterLabelGO+ model). Furthermore, the complementary performance of the alignment-based method AlignmentKNN suggests that combining alignment-based and neural network-based approaches can lead to further improvements in protein function prediction.

### 3.2 Comparison of different loss functions and their combinations

To investigate whether the use of custom loss functions contributes to the success of InterLabelGO and to examine the effect of using different loss functions on the final performance, we trained different models with various loss functions and their combinations on the training dataset mentioned in Supplementary Table S1. The loss functions considered in here include the ZLPR loss, BCE loss, protein-centric F1 loss (PTF1), and GO-centric F1 loss (GOF1). The combinations of these loss functions were obtained by simply multiplying them together. The performance of these models was then evaluated using the test set described in Supplementary Table S1 and presented in Supplementary Table S3.

Our results show that adding PTF1 or GOF1 to BCE improved performance by mitigating label imbalance. ZLPR loss outperformed BCE, with further enhancements when combined with PTF1 and GOF1. Interestingly, the PTF1 and GOF1 losses, when used independently, perform poorly compared to other loss configurations. This observation suggests that these losses, while effective in addressing label imbalance, may not be sufficient on their own to capture the complex relationships between GO terms and protein sequences. The combination of these losses with ZLPR or BCE appears to be crucial for achieving optimal performance.

Among the various loss combinations, the two best combinations are ZLPR_GOF1 and ZLPR_PTG1_GOF1 which perform similarly to each other. However, we ultimately selected ZLPR_PTF1_GOF1 as the primary loss configuration for training InterLabelGO. This decision was based on its superior overall performance when combined with AlignmentKNN according to the validation dataset. The combination of ZLPR, PTF1, and GOF1 likely strikes a balance between capturing the pairwise ranking of GO terms, addressing label imbalance, and considering both protein-centric and GO-centric perspectives.

Our study highlights the critical role of loss function selection in protein function prediction. The combination of ZLPR, PTF1, and GOF1 effectively addresses label imbalance and captures complex GO term relationships. The superior performance of ZLPR_PTF1_GOF1 with AlignmentKNN demonstrates its potential for robust and accurate predictions in real-world scenarios.

### 3.3 Advantage of hybrid model

To demonstrate the advantages of the hybrid approach employed by InterLabelGO+, we conducted a case study on three representative proteins from our test dataset, each with varying levels of sequence similarity to proteins in the GOA template database: Transcriptional regulatory protein dep1 (UniProt ID: Q9P7M1) from *Schizosaccharomyces pombe* (fission yeast), Glutamate receptor interacting protein 2 isoform X3 (UniProt ID: A0A8M6Z252) from *Danio rerio* (Zebrafish), and DEAD/H (Asp-Glu-Ala-Asp/His) box polypeptide 20 (UniProt ID: A6K3R6) from *Rattus norvegicus* (Rat). These proteins have 17, 25, and 32 experimentally annotated GO terms, respectively, under the BPO aspect, with all parent terms being propagated through “is-a” and “part-of” relationships. Supplementary Table S4 compares the performance of InterLabelGO+ with other methods for BPO prediction across various proteins. The number in parentheses next to each protein UniProt ID indicates the mean top five sequence similarity based on DIAMOND hits. For each method, the prediction score threshold is set to the value that achieved the best *F*_max_ on the entire test dataset. The best-performing method in each category is highlighted in bold.

**Figure 3.**
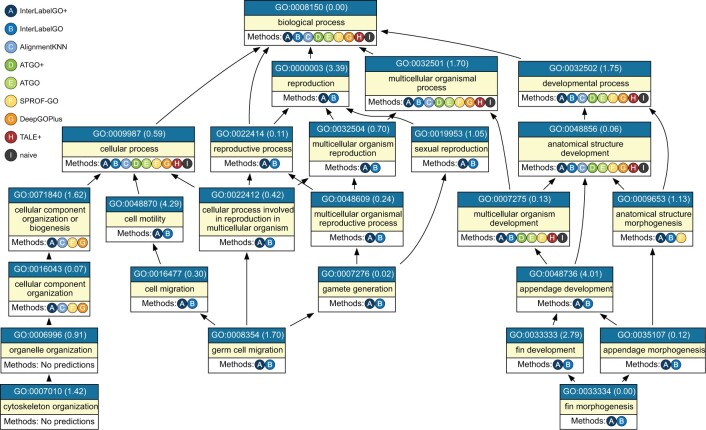
Directed acyclic graph showing the biological process GO terms for the representative example A0A8M6Z252. All methods that correctly predicted each GO term are listed below the corresponding term. The number in parentheses next to each GO term represents its IA value.

Figure 3 shows the DAG of GO terms for protein A0A8M6Z252, with the IA value in parentheses. The methods that correctly predicted each term are listed below it.

The case studies results reveal the strengths of InterLabelGO and InterLabelGO+ in capturing complex relationships between GO terms and predicting groups of related terms across various sequence similarity scenarios.

For the challenging protein Q9P7M1, which lacks significant hits in the GOA template database, InterLabelGO demonstrates its promise by maintaining relatively good performance compared to all other methods. When considering composite methods that combine alignment-based and deep learning approaches, InterLabelGO+ still performs the best. Unlike ATGO+ and ATGO, where the combination of deep learning and alignment-based methods can lead to decreased performance if the alignment-based component fails to provide reliable predictions, the dynamic weighting scheme in InterLabelGO+ helps mitigate the negative impact on the overall results.

For A0A8M6Z252, where AlignmentKNN has hits but produces many false positive predictions, InterLabelGO+ demonstrates the complementary nature of InterLabelGO and AlignmentKNN. This combination effectively filters out most false positives from AlignmentKNN while incorporating true positives missed by InterLabelGO, further improving overall performance.

For A6K3R6, where the GOA template database contains very similar proteins, AlignmentKNN performs exceptionally well, surpassing all other methods, including InterLabelGO and the composite method TALE+. The combination of alignment-based predictions and InterLabelGO in InterLabelGO+ maintains the high true positive rate from AlignmentKNN while reducing false positives, again demonstrating the complementary nature of these approaches and the importance of the hybrid approach.

Further analysis of the case study for protein A0A8M6Z252 (Fig. 3) reveals the specific advantages of InterLabelGO+. The model’s ability to capture label dependencies and address label imbalance through its composite loss function led to improved prediction accuracy, especially for high IA terms. For instance, InterLabelGO+ correctly predicted terms such as GO: 0000003 (reproduction, IA = 3.39) and GO: 0048870 (cell motility, IA = 4.29), which were missed by several other methods. An in-depth analysis of the label correlations and the effects of different loss functions on prediction accuracy is provided in Supplementary Section S2. Additionally, the integration of AlignmentKNN in InterLabelGO+ allowed for successful prediction of terms like GO: 0071840 and GO: 0016043, which were missed by InterLabelGO alone, highlighting the benefits of combining deep learning and alignment-based approaches.

## 4 Discussion

InterLabelGO is a novel alignment-free deep learning-based approach that accurately predicts protein functions from sequence information alone. Our model addresses several challenges in protein function prediction, including label imbalance and label dependencies, by incorporating a composite loss function that combines a GO term-aware weighted F1 loss and a pairwise ranking-based loss. This enables InterLabelGO to capture complex functional relationships and mitigate the impact of skewed functional annotation distributions. Furthermore, InterLabelGO+ integrates sequence homology-based predictions through a dynamic weighting scheme, leveraging complementary information to enhance its predictive performance. The superior performance of InterLabelGO+ compared to state-of-the-art methods across various functional categories and evaluation metrics demonstrates its effectiveness in unraveling the functional landscape of proteins. In addition, we made use of the CAFA5 late submission evaluation system to determine how InterLabelGO+ would have performed in the competition; through this itnerface, InterLabelGO+ achieved an average *wF*_max_ of 0.5764, which would have placed in third overall. This performance is significantly higher than the CAFA5 version of InterLabelGO (*wF*_max_ of 0.5604), due to the incorporation of the dynamic weighting scheme in our final version of the pipeline. The strong performance of InterLabelGO+ in the CAFA challenge underscores its potential for real-world applications and its competitiveness among top-performing methods in the field.

In future work, we plan to explore additional features and architectures to enhance InterLabelGO+. One promising approach is incorporating attention mechanisms, which have shown success in natural language processing and protein function prediction as demonstrated by SPROF-GO (Yuan *et al.* 2023). By integrating attention mechanisms, we aim to enable InterLabelGO+ to focus on informative sequence regions, potentially improving both predictive performance and model interpretability by highlighting key patterns contributing to specific functions. Furthermore, we plan to extend the deep learning model of InterLabelGO to handle multi-modal data, such as protein-protein interaction (PPI) networks and literature-derived features. PPI data provides valuable information about functional associations between proteins, while literature-derived features capture relevant functional information from scientific literature. Techniques like BioBERT (Lee *et al.* 2020) and the approach in DeepText2GO (You *et al.* 2018) could be used to extract meaningful features from the literature. By incorporating these additional data sources, we aim to provide InterLabelGO+ with a more comprehensive understanding of protein functions and further improve its predictive capabilities.

## Author contributions

Conceptualization & Supervision, C.Z. and L.F.; Funding Acquisition: L.F.; Methodology, Q.L.; Formal analysis, Q.L., C.Z. and L.F.; Web-server: C.Z.; Writing—Original Draft, Q.L.; Writing—Review & Editing, C.Z. and L.F.

## Supplementary Material

btae655_Supplementary_Data

## Data Availability

All data and source code used in the present study is available at https://github.com/QuanEvans/InterLabelGO. FASTA files of the training, validation, and testing sequences, along with the corresponding Gene Ontology labels, are available at https://seq2fun.dcmb.med.umich.edu/InterLabelGO.
