# Supplementary material for: InterLabelGO+: unraveling label correlations in protein function prediction
Source: Bioinformatics. 2024 Nov 5;40(11):btae655. doi: 10.1093/bioinformatics/btae655 (PMC11568131; doi:10.1093/bioinformatics/btae655)
Supplement: btae655_Supplementary_Data [file btae655_supplementary_data.pdf]

Supplementary materials for InterLabelGO+: Unraveling label correlations in protein function prediction

1. EVALUATION

To evaluate our predictions, we adopted the CAFA (Zhou *et al.*, 2019) challenge metrics, specifically the maximum weighted F-measure (wF_{max}) and the minimum semantic distance (S_{min}) (Clark and Radivojac, 2013). In addition, we report the area under the weighted precision-recall curve(AUWPR), which is particularly relevant for datasets with significant label imbalance as it places greater emphasis on correctly predicting minority classes (Davis and Goadrich, 2006). The AUWPR is calculated by plotting the weighted precision (wpr) against the weighted recall (wr) at various threshold settings and computing the area under the resulting curve where the weighted precision and recall are defined in **Equation S3**.

A. Weighted F-measure (wF_{max})

The metric wF_{max} represents the maximum of the protein-centric information accretion-weighted F-measure across all prediction thresholds τ. It is formulated as:

$$\text{wpr}(\tau) = \frac{1}{m(\tau)} \sum_{i=1}^{m(\tau)} \frac{\sum_q \text{IA}(q) \cdot \mathbb{1}(q \in P_i(\tau) \wedge T_i)}{\sum_q \text{IA}(q) \cdot \mathbb{1}(q \in P_i(\tau))}, \tag{S1}$$

$$\text{wrc}(\tau) = \frac{1}{n} \sum_{i=1}^{n_e} \frac{\sum_q \text{IA}(q) \cdot \mathbb{1}(q \in P_i(\tau) \wedge T_i)}{\sum_q \text{IA}(q) \cdot \mathbb{1}(q \in T_i)}, \tag{S2}$$

$$wF_{\text{max}} = \max_{\tau \in (0,1]} \left(\frac{2 \cdot \text{wpr}(\tau) \cdot \text{wrc}(\tau)}{\text{wpr}(\tau) + \text{wrc}(\tau)} \right) \tag{S3}$$

In this context, $\text{wpr}(\tau)$ and $\text{wrc}(\tau)$ denote the information accretion-weighted precision and recall at a given prediction score threshold τ. $P_i(\tau)$ is the set of predicted terms for a protein i with a score equal to or higher than τ, and T_i represents the set of true terms for protein i. The number $m(\tau)$ indicates the count of sequences with at least one predicted score meeting or exceeding τ, and n is the total number of proteins in the benchmark. q represents the q-th GO term and $\text{IA}(q)$ represents the information accretion of the q-th GO term as defined in the **Equation 1** in the main text.

B. Minimum Semantic Distance (S_{min})

The metric S_{min} calculates the semantic distance between actual and predicted annotations, considering the information accretion of classes. It is defined as:

$$\text{ru}(\tau) = \frac{1}{n} \sum_{i=1}^{n} \sum_q \text{IA}(q) \cdot \mathbb{1}(q \notin P_i(\tau) \wedge q \in T_i), \tag{S4}$$

$$\text{mi}(\tau) = \frac{1}{n} \sum_{i=1}^{n} \sum_q \text{IA}(q) \cdot \mathbb{1}(q \in P_i(\tau) \wedge q \notin T_i), \tag{S5}$$

$$S_{\text{min}} = \min_{\tau \in (0,1]} \left(\sqrt{\text{ru}(\tau)^2 + \text{mi}(\tau)^2} \right) \tag{S6}$$

Here, $\text{ru}(\tau)$ represents the remaining uncertainty, and $\text{mi}(\tau)$ represents the missing information at threshold τ. For other terms, please refer to the **Section A** for their definitions.

Table S1. Number of unique terms across different aspects for the training, validation, and test sets.

	Training			Validation			Testing		
**Method**	BPO	CCO	MFO	BPO	CCO	MFO	BPO	CCO	MFO
Numbers of proteins	92382	92920	78607	1146	699	563	5819	3440	2080
Numbers of annotations	3503437	1196126	669976	25252	7357	4081	169459	41592	19773
Numbers of unique terms	21321	2957	7224	3108	342	716	5781	689	1400

Table S2. Comparison of performance on the Uniprot-GOA 20230316-20240209 release.

	$\mathbf{wF_{max}}$			$\mathbf{S_{min}}$			**AUWPRC**		
**Method**	BPO	CCO	MFO	BPO	CCO	MFO	BPO	CCO	MFO
InterLabelGO+	**0.687**	**0.748**	**0.748**	**13.398**	**4.686**	**4.867**	**0.516**	**0.709**	**0.550**
InterLabelGO	0.681	0.735	0.723	13.593	4.818	5.286	0.441	0.624	0.479
AlignmentKNN	0.613	0.700	0.723	16.231	5.272	5.116	0.451	0.637	0.515
ATGO+	0.554	0.665	0.691	17.120	5.653	5.805	0.387	0.625	0.369
ATGO	0.551	0.664	0.688	16.879	5.682	5.787	0.397	0.660	0.377
SPROF-GO	0.468	0.647	0.641	21.512	5.960	7.007	0.377	0.678	0.477
DeepGOPlus	0.557	0.658	0.672	17.038	6.133	5.944	0.354	0.527	0.375
TALE+	0.502	0.614	0.661	20.594	6.527	6.558	0.406	0.632	0.489
naïve	0.226	0.340	0.186	27.968	9.194	12.550	0.110	0.300	0.087

Best performance in bold; for wF_{max} and AUWPRC this is the highest value; for S_{min} it is the lowest.

Table S3. Performance comparison of different loss functions and their combinations on the UniProt-GOA 20230316-20240209 release.

	$\mathbf{wF_{max}}$			$\mathbf{S_{min}}$			**AUWPRC**		
**Method**	BPO	CCO	MFO	BPO	CCO	MFO	BPO	CCO	MFO
ZLPR_PTF1_GOF1	0.681	**0.735**	0.723	13.593	4.818	5.286	0.441	0.624	0.479
ZLPR_GOF1	**0.682**	0.730	**0.729**	**13.196**	4.830	**5.213**	0.427	0.648	0.481
ZLPR_PTF1	0.665	0.732	0.726	17.568	4.950	5.332	0.440	0.626	0.478
ZLPR	0.677	0.734	0.726	13.910	**4.730**	5.282	0.441	0.650	0.485
BCE_ZLPR	0.674	0.731	0.728	13.942	4.768	5.249	0.443	0.652	0.486
BCE_PTF1_GOF1	0.661	0.717	0.684	13.914	5.142	6.115	0.464	0.620	0.500
BCE_PTF1	0.647	0.713	0.695	14.690	5.158	5.873	**0.466**	0.619	0.499
BCE_GOF1	0.642	0.706	0.702	14.037	5.183	5.735	0.434	0.674	0.505
BCE	0.627	0.701	0.694	14.785	5.258	5.855	0.446	**0.675**	**0.515**
PTF1_GOF1	0.423	0.587	0.488	220.568	7.371	9.723	0.179	0.241	0.162
PTF1	0.306	0.598	0.492	419.441	7.322	13.357	0.086	0.236	0.139
GOF1	0.139	0.247	0.265	29.689	9.007	10.700	0.062	0.167	0.095

Best performance in bold. wF_{max} and AUWPRC, highest; S_{min}, lowest.

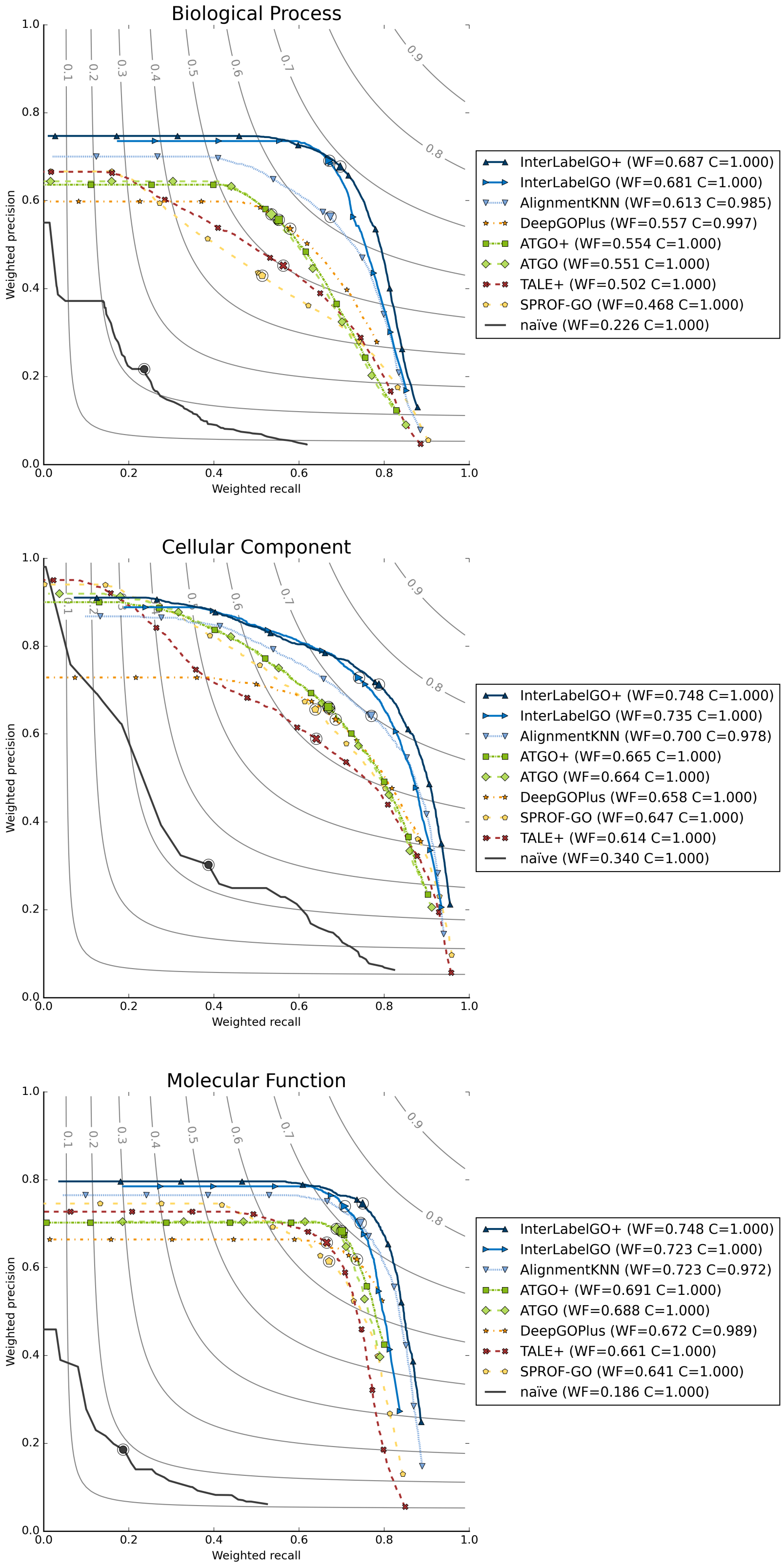

Fig. S1. Weighted precision-recall curves for top-performing methods across the three GO ontologies based on the UniprotGOA 202303-202402 release. The ideal performance (w$F_{\max}$ = 1) is at the top right. Dots indicate the maximum w$F_{\max}$. The legend shows the w$F_{\max}$ values (WF) and the fraction of proteins predicted by each method (coverage; C).

Table S4. Performance comparison of InterLabelGO/InterLabelGO+ and seven other methods for BPO prediction on three representative proteins. Mean top 5 sequence similarity by DIAMOND is in parentheses.

	Q9P7M1 (N/A)				A0A8M6Z252 (0.61)				A6K3R6 (0.84)			
Method	F1-score	FN	FP	TP	F1-score	FN	FP	TP	F1-score	FN	FP	TP
InterLabelGO+	**0.774**	5	2	12	**0.939**	**2**	1	**23**	**0.955**	**0**	3	**32**
InterLabelGO	0.741	7	**0**	10	0.913	4	**0**	21	0.731	13	**1**	19
AlignmentKNN	0.000	17	0	0	0.177	18	47	7	0.853	0	11	32
ATGO+	0.516	9	6	8	0.324	19	6	6	0.356	24	5	8
ATGO	0.562	8	6	9	0.324	19	6	6	0.391	23	5	9
TALE+	0.364	9	19	8	0.250	19	17	6	0.831	0	13	32
SPROF-GO	0.395	**2**	44	**15**	0.290	16	28	9	0.487	13	27	19
DeepGOPlus	0.000	17	0	0	0.389	18	4	7	0.615	0	40	32
naïve	0.250	13	11	4	0.300	19	9	6	0.170	28	11	4

Best performance in bold. F1-score, TP and FN, highest; FP, lowest.

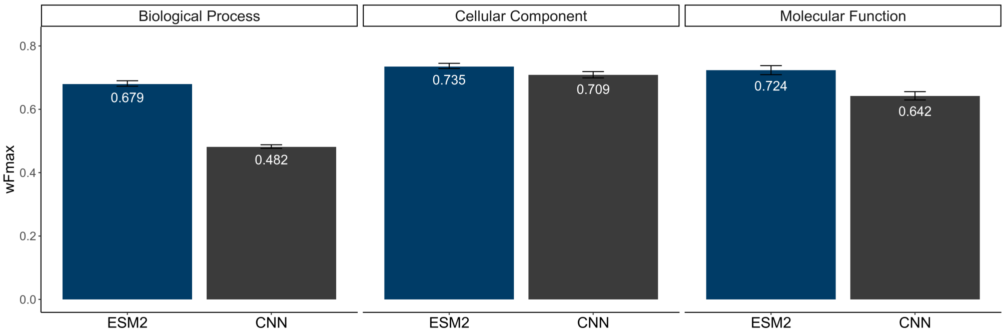

Fig. S2. Performance comparison of InterLabelGO using ESM2 vs CNN features. To quantify the contribution of ESM2 (Verkuil *et al.*, 2022) to the InterLabelGO deep learning component, we compared the performance of our model using ESM2 features against a version using a Convolutional Neural Network (CNN) with one-hot encoding for sequence representation. Both models used the same loss function (ZLPR_PTF1_GOF1) to ensure a fair comparison.

2. DETAILED ANALYSIS OF CASE STUDY (A0A8M6Z252)

To better understand the superior performance of InterLabelGO for protein A0A8M6Z252, we conducted an in-depth analysis of different loss functions and their impact on prediction accuracy. The performance of various loss functions on protein A0A8M6Z252 was evaluated using the metrics of True Positives (TP), False Positives (FP), False Negatives (FN), and Weighted F1-score in **Table** S5. These results demonstrate a substantial improvement when using ZLPR over BCE, with further refinement when incorporating the IA-weighted protein-centric and GO-centric F1 losses.

Additionally, we analyzed the correlations between parent GO terms under two specific branches: GO:0008354 (germ cell migration) and GO:0033334 (fin morphogenesis). Table S6 shows the conditional probabilities between selected GO terms under these branches, calculated using the training dataset. Notably, we observed high correlations across these branches. For instance, multicellular organism development (GO:0007275) from the fin morphogenesis branch and cell motility (GO:0048870) from the germ cell migration branch show a notable correlation, with P(GO:0007275 | GO:0048870) = 0.611. These terms provide an important example of the

general principle that the high degree of mutual information between terms in the GO hierarchy is better captured by the model trained using the ZLPR loss function.

Table S5. Performance comparison of different loss functions for protein A0A8M6Z252.

Method	TP	FP	FN	Weighted F1-score
BCE	19	3	6	0.846
ZLPR	**25**	2	**0**	0.909
ZLPR_PTF1_GOF1	21	**0**	4	**0.924**

Best performance in bold. Weighted F1-score, TP and FN, highest; FP, lowest.

Table S6. Conditional probabilities between selected parent GO terms under branches of germ cell migration (GO:0008354) and fin morphogenesis (GO:0033334)

GO Term A	GO Term B	P(A ∣ B)	P(B ∣ A)
GO:0009653	GO:0007275	0.501	0.852
GO:0009653	GO:0048609	0.354	0.098
GO:0009653	GO:0022412	0.475	0.103
GO:0009653	GO:0048870	0.498	0.130
GO:0007275	GO:0048609	0.546	0.089
GO:0007275	GO:0022412	0.598	0.076
GO:0007275	GO:0048870	0.611	0.093
GO:0048609	GO:0022412	0.887	0.693
GO:0048609	GO:0048870	0.187	0.175
GO:0022412	GO:0048870	0.168	0.202

REFERENCES

Clark, W. T. and Radivojac, P. (2013). Information-theoretic evaluation of predicted ontological annotations. *Bioinformatics*, 29(13):i53–i61.

Davis, J. and Goadrich, M. (2006). The relationship between Precision-Recall and ROC curves. In *Proceedings of the 23rd International Conference on Machine Learning*, pages 233–240.

Verkuil, R., Kabeli, O., Du, Y., *et al.* (2022). Language models generalize beyond natural proteins. *bioRxiv*, pages 2022–12.

Zhou, N., Jiang, Y., Bergquist, T. R., *et al.* (2019). The CAFA challenge reports improved protein function prediction and new functional annotations for hundreds of genes through experimental screens. *Genome Biology*, 20:1–23.
